# Spectrum of Pompe disease in childhood – The Toronto experience

**DOI:** 10.1186/1471-2474-14-S2-P3

**Published:** 2013-05-29

**Authors:** J Raiman, G Bendiak, S Hewson, M Mecija, I Narang, M Saunders

**Affiliations:** 1 Division of Clinical & Metabolic Genetics, Hospital for Sick Children, Toronto, Ontario, Canada; 2 Division of Respiratory Medicine, Hospital for Sick Children, Toronto, Ontario, Canada

## Introduction

Pompe disease, caused by the deficiency of acid alpha glucosidase [GAA], ranges from the severe infantile to attenuated adult forms. We present clinical features and treatment outcomes from 4 paediatric cases from our centre in the last 4 years.

## Results

1) A 4-month-old female presented with hypotonia and cardiomegaly. She was CRIM negative and homozygous for p.R854X. She successfully received immunotolerance induction therapy (ITI) prior to ERT at 4.5 months. She remained antibody negative until 12 months, when titers rose to 1:800. Hex4 increased from 48.4 at baseline to 108.8. LVMI decreased from 220 at baseline to 119 g/m2. She started nocturnal BiPAP at 11 months, then developed pneumonia and became ventilator dependent, leading to cessation of ERT and death at 14 months.

2) A male infant born at 30-weeks was investigated for hypertrophic cardiomyopathy and skeletal myopathy. He was diagnosed at 4 months, was CRIM negative, and homozygous for c 546+2 T>C . He received ITI, started ERT at 4.1 months, and remains antibody negative. Nocturnal BiPAP was started at 5 months. Hex4 dropped from 59.7 at baseline to 54.9 at the last follow up. LVMI decreased from 445 at baseline to 101.7 at 24 months. He remains on ERT at 24 months and has achieved minor motor gains.

3) A 6-month-old male infant presented with cardiomyopathy and skeletal myopathy and was BiPAP dependent. He was CRIM negative and had a c.2544delC / complex deletion. The family elected not to pursue ERT and he died at 7 months.

4) A 25-month-old male presented with recurrent respiratory infections, proximal limb weakness, ventricular hypertrophy and speech delay. He was CRIM positive and heterozygous for p.P361L/p.R854X. He started ERT at 23 months. Hex4 dropped from 195.5 at baseline to 70. Positive antibody titers of 1:200 at 2 months rose to 1:800 after 15 months. He remains on ERT after 18 months with motor stability and has made developmental gains.

## Discussion

The outcomes of our cases highlight the impact of earlier diagnosis and treatment and the need for multidisciplinary support (particularly respiratory). Pre ERT ITI was well tolerated.

